# *Ex-utero* third trimester developmental changes in functional brain network organization in infants born very and extremely preterm

**DOI:** 10.3389/fnins.2023.1214080

**Published:** 2023-09-01

**Authors:** Kevin M. Cook, Josepheen De Asis-Cruz, Sudeepta K. Basu, Nickie Andescavage, Jonathan Murnick, Emma Spoehr, Adré J. du Plessis, Catherine Limperopoulos

**Affiliations:** ^1^Developing Brain Institute, Children’s National Hospital, Washington, DC, United States; ^2^Department of Diagnostic Imaging & Radiology, Children’s National Health System, Children’s National Hospital, Washington, DC, United States; ^3^Prenatal Pediatrics Institute, Children’s National Hospital, Washington, DC, United States

**Keywords:** graph theory, resting state fMRI, brain development, prematurity, stress, brain network

## Abstract

**Introduction:**

The latter half of gestation is a period of rapid brain development, including the formation of fundamental functional brain network architecture. Unlike *in-utero* fetuses, infants born very and extremely preterm undergo these critical maturational changes in the extrauterine environment, with growing evidence suggesting this may result in altered brain networks. To date, however, the development of functional brain architecture has been unexplored.

**Methods:**

From a prospective cohort of preterm infants, graph parameters were calculated for fMRI scans acquired prior to reaching term equivalent age. Eight graph properties were calculated, Clustering Coefficient (C), Characteristic Path Length (L), Modularity (Q), Local Efficiency (LE), Global Efficiency (GE), Normalized Clustering (λ), Normalized Path Length (γ), and Small-Worldness (σ). Properties were first compared to values generated from random and lattice networks and cost efficiency was evaluated. Subsequently, linear mixed effect models were used to assess relationship with postmenstrual age and infant sex.

**Results:**

A total of 111 fMRI scans were acquired from 85 preterm infants born at a mean GA 28.93 ± 2.8. Infants displayed robust small world properties as well as both locally and globally efficient networks. Regression models found that GE increased while L, Q, λ, γ, and σ decreased with increasing postmenstrual age following multiple comparison correction (r^2^_Adj_ range 0.143–0.401, *p* < 0048), with C and LE exhibited trending increases with age.

**Discussion:**

This is the first direct investigation on the extra-uterine formation of functional brain architecture in preterm infants. Importantly, our results suggest that changes in functional architecture with increasing age exhibit a different trajectory relative to *in utero* fetus. Instead, they exhibit developmental changes more similar to the early postnatal period in term born infants.

## 1. Introduction

The late second and third trimesters of pregnancy are a period of rapid brain development. Fetuses experience exponential increases in brain volume (Andescavage et al., 2017) and are developing the earliest functional proto-networks (van den Heuvel and Thomason, 2016; Turk et al., 2019) throughout the gestational period leading up to birth at term-age. However, infants born very and extremely preterm (V/EPT) at less than 32- and 28-weeks postmenstrual age (PMA) respectively, experience these ‘trimesters’ under stress of premature transition to the extra-uterine environment, sensory stimulation, and dependency on life-support interventions. These earlier born infants spend this period outside of the shielded intrauterine milieu, exposed to the more sensorially intense extrauterine world in a hostile neonatal intensive care environment. Although there is evidence that early extrauterine exposure is associated with atypical brain growth and functional connectivity, it remains unexplored how this exposure alters the developmental trajectory of the emerging functional brain architecture. Such studies are necessary to understand the implications of premature birth on functional brain development.

During typical intrauterine development, fetuses exhibit significant increases in global, regional and tissue-specific brain volumetric growth (Carne et al., 2006; Andescavage et al., 2017; Wu et al., 2020) and the emergence of prominent white matter tracks (Jaimes et al., 2020) during the second half of pregnancy. Alongside structural development, this is also a crucial period for functional brain development. During this period we can observe the organization and emergence of proto-networks (van den Heuvel and Thomason, 2016; Turk et al., 2019), as well as the core functional brain architecture. More recently, graph theoretic techniques have been applied to functional brain networks as a means to quantitively model their complex network structures and interactions (Bullmore and Sporns, 2009; Rubinov and Sporns, 2010). Using a construction of regions (nodes) and their connections (edges), a graph approach allows for the modeling of the overall network architecture and organization. During the fetal period, graph approaches have been used to showcase lobular functional maturation (Jakab et al., 2014), sensorimotor and association area hubs (van den Heuvel et al., 2018; Turk et al., 2019), and increasing modular integration with increasing age (Thomason et al., 2014). Importantly, prior work in fetuses from 19–40 weeks’ gestational age have shown that during the second half of pregnancy there is small-world topology and that measures related to network segregation decreased with advancing gestational age (De Asis-Cruz et al., 2021).

Of note, little is known about the development of this critical architecture during the *ex-utero*, preterm period, despite clear evidence that brain structure (Matthews et al., 2018; Loh et al., 2020; Wu et al., 2020) and functional networks (He and Parikh, 2016; Rogers et al., 2017; Wheelock et al., 2021) differ between infants born at term and those born preterm. A single study has attempted to examine differences in graph properties during a developmental period in *ex-utero* infants ranging from 31–42 weeks from Cao et al. (2017). While their findings suggest that increasing segregation can be observed with increasing ages, the study cohort was comprised of a mixed sample of term and preterm infants. Subsequent work comparing term-born and E/VPT infants at term equivalent age (TEA), however, has found that term-born infants exhibit significant differences in network architecture compared to V/EPT infants. Specifically, V/EPT infants at TEA exhibit decreased clustering coefficients as well as local and global efficiency, while also exhibiting greater characteristic path lengths suggesting significantly diminished segregation and integration in E/VPT relative to term-born infants (Bouyssi-Kobar et al., 2019). Finally, although the impact of prematurity on graph metrics have not been directly investigated, we have observed differences in functional connections in preterm infants compared to age-matched *in-utero* fetuses (De Asis-Cruz et al., 2020). These differences at TEA suggest that there is a fundamental difference in the developmental trajectories of infants born V/EPT relative to healthy born term infants.

Given differences in brain development between infants born at term and those born preterm at term-equivalent age, there is a significant gap in our understanding of how functional brain architecture changes across the *ex-utero* preterm period as experienced by V/EPT infants. In a cohort of preterm infants born before 32 weeks gestational age, we examined changes in functional network architecture between 25–40 weeks. Using a graph theoretic approach, we aim to characterize how the functional brain organization changes over time to assess how earlier-than-typical exposure to the extrauterine environment may be associated with atypical development.

## 2. Materials and methods

### 2.1. Participants

A total of 365 preterm age scans were collected from a cohort of 226 V/EPT infants which were recruited for a prospective cohort study at Children’s National Hospital. Infants underwent 1–3 scans prior to TEA depending on their relative age at birth and stability (mean = 2.12). Of the collected scans, 152 were excluded due to infants exhibiting more than mild structural brain injury based on their Kidokoro score (Kidokoro et al., 2013), leaving a total of 204scans from 126infants. Of these, an additional 93 scans were excluded for exceeding motion criteria described below, yielding a total of 111 fMRI scans from 85 infants. Within the final sample, infants were born at a mean GA of 28.76 weeks and scanned at a mean PMA of 33.43 weeks (Table 1).

**Table 1:** Sample demographics and clinical characteristics.

	Summary	Range
Mean birth GA (weeks)	28.76 (2.47)	[24–32.88]
Mean scan PMA (weeks)	33.43 (2.65)	[25.72–39.86]
Mean age (weeks)	4.55 (2.99)	[0.71–12.71]
Sex (Male)	49 (44.14%)	
Median APGAR		
1 Minute	4	[1, 9]
5 Minute	8	[1, 9]
Delivery (Vaginal)	36 (42.35%)	
Birth weight (g)	1222.8 (461.6)	[464, 2,600]
Head circumference (cm)	25.9 (3.3)	[11.4, 33]
Bronchopulmonary dysplasia	36 (42.35%)	
Retinopathy of prematurity	64 (75.29%)	
Sepsis	8 (10.59%)	
Kidokoro injury scores		
None	67 (60.36%)	
Mild	44 (39.36%)	
IVH Grade 1	20 (45.45%)	
IVH Grade 2	24 (54.55%)	

GA, Gestational Age; PMA, Post Menstrual Age, IVH, Intraventricular Hemorrhage Grade.

### 2.2. Image acquisition and preprocessing

All MRI images were acquired using a 1.5 T GE scanner (Discovery MR450, GE Healthcare, Milwaukee, WI) using an 8-channel infant head coil. To minimize motion, infants were fed prior to scanning, swaddled in a warm blanket, secured using an infant vacuum pillow, and provided with ear protection consisting of silicone ear plugs and adhesive earmuffs. Infants were not otherwise sedated. Anatomical T2 weighted 3D fast-spin echo structural images were collected with the following parameters: 2502 ms TR, 84.37 ms TE, 0.5 × 0.5 × 0.5 mm voxel size, 90° flip angle. Resting state echo planar images (EPI) were collected with: 2000 ms TR, 30 ms TE, 2.3 × 2.3 × 4.0 mm voxel size, 60° flip angle, 80 cm field of view, and 10 min scan duration (300 volumes).

Resting state data were preprocessed using previously published pipelines (De Asis-Cruz et al., 2018) using tools from the Analysis of Functional NeuroImages (AFNI) (Cox, 1996). In brief, scans underwent within-volume motion correction, slice-time correction, dropping the first four volumes. Following, images were despiked, bias-field corrected, and outliers were censored. Functional images were then motion corrected, aligned with their anatomical, and normalized to an age matched fetal template (Gholipour et al., 2017). Signal was intensity scaled to a global mode of 1,000 and smoothed using a 5 mm full-width half-maximum blur. Demeaned motion was calculated and volumes exceeding 0.2 mm were excluded. Motion, CSF, and white matter signal were regressed out, and the image underwent 0.009–0.08 bandpass filtering. After preprocessing, only images with at least 4 min of available data were included in the analyses.

### 2.3. Analysis strategy

Time series data was collected from regions within the infant aal atlas (Shi et al., 2011) warped to the gestational age templates with additional ROIs for the left and right cerebellum and brainstem. Subsequently, Pearson correlations were performed between each ROI and then z-transformed resulting in a 93×93 connectivity matrix. Using these matrices and the Brain Connectivity Toolbox (Rubinov and Sporns, 2010), graph theory metrics were then computed. To do this, metrics were computed for each infant’s 93×93 binary, undirected graph at each interval, over the thresholding of 0.10–0.41 based on thresholding of individual networks using correlation strengths for each respective network, alongside 100 random and lattice networks which preserve degree distribution. Correlation-based thresholding was utilized in lieu of density-based to minimize distortions found to be common in utilizing density based approaches in neuroimaging data (Hallquist and Hillary, 2019). For all analyses except cost analyses, the mean value for each individual was averaged across cost thresholds (Lynall et al., 2010).

The resulting paraments included Clustering Coefficient (C), or the likelihood for neighbor nodes to be connected to each other; Modularity (Q), or the tendency for nodes to form smaller subnetworks; and Local Efficiency (LE), or the ease of information to pass between nearby nodes. These three parameters represent the overall network segregation, or the degree to which the functional architecture is organized into specialized units. We also calculated Characteristic Path Length (L), or the shortest average distances between two nodes; and Global Efficiency (GE), or the ease of which information passes through the entire network. These two parameters represent the overall network integration, or the degree to which architecture is organized in a manner to facilitate information flow throughout the network. Finally, we calculated measures of Normalized Clustering (γ), Normalized Path Length (λ), and Small-Worldness (σ) to evaluate small-world topology. Subsequently, random and lattice networks were simulated, with random graphs representing an unstructured and irregular network and lattice graphs representing a highly structured and regular network, from which network properties were then also calculated.

First, following calculation of graph theoretic properties, the initial 5 values were compared to their random and lattice network estimates via multiple ANOVAs. Subsequently, post-hoc differences were assessed via Tukey test (Tukey, 1977). Subsequently, the small world topology of these networks were evaluated by subjecting σ to independent one-sample t-tests to evaluate whether the mean values were greater than 1.0 and thus exhibiting small world topology (Humphries and Gurney, 2008). Given the variable in motion across infants, a linear mixed effect model controlling for the repeated measurements within infants and postmenstrual age was utilized to test if there were significant differences between infants with high degrees of motion censoring against those with a low number of censored volumes.

Second, efficiency and cost of networks were evaluated by characterizing the individuals mean GE and LE to their random and lattice networks values across each of the thresholds (costs). Subsequently, cost efficiency (CE) was determined by subtracting the cost from each mean GE at that cost.

Third, we assessed differences in graph properties over time. Using a linear mixed effect model to control for the repeated measurements within infants, postmenstrual age and sex were used as predictors for each graph metric independently.

## 3. Results

### 3.1. Network structure in preterm infants

**Figure 1:**
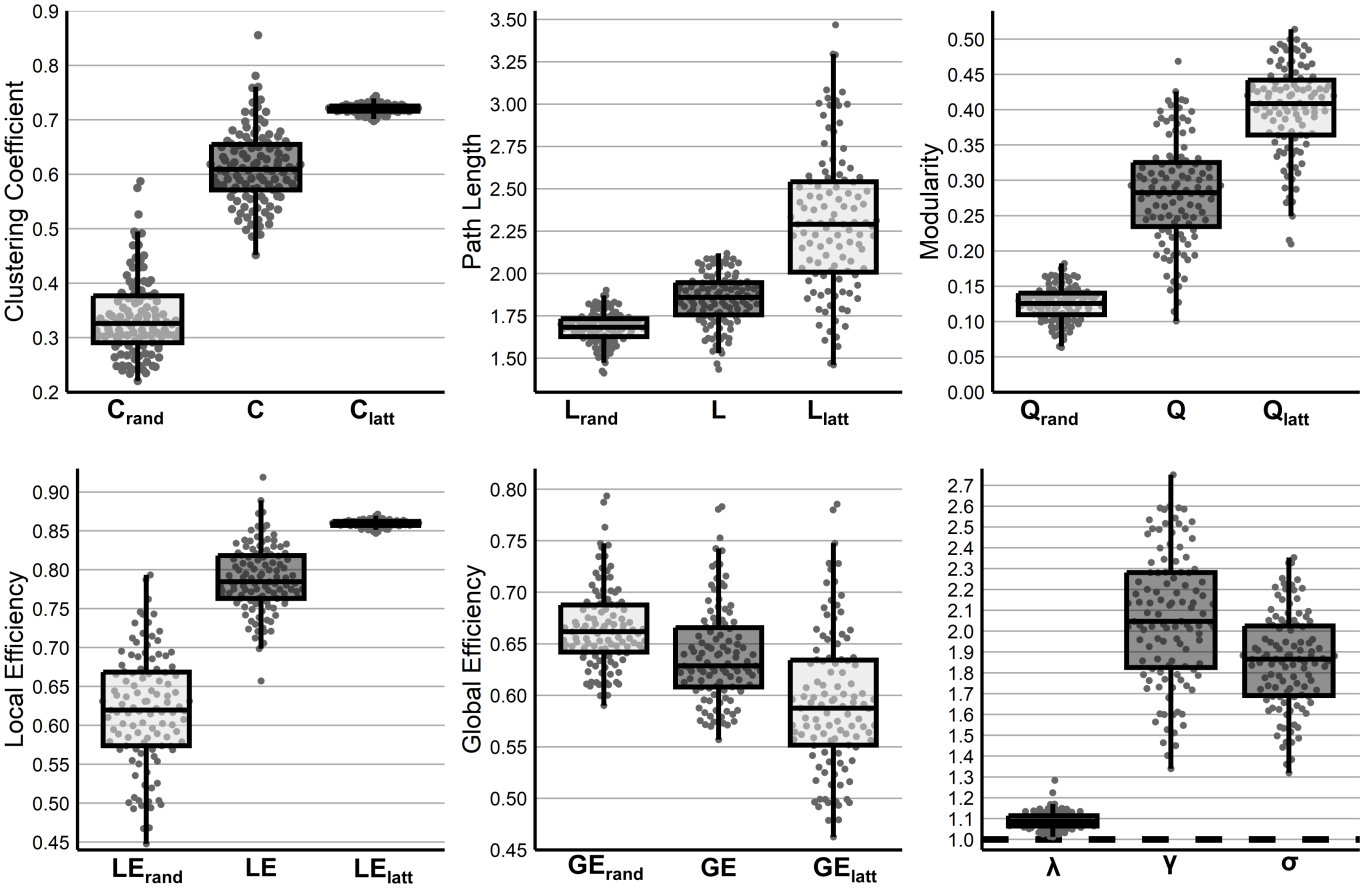
Network features of the preterm functional connectome. Global network properties across infants compared to their random and lattice networks (C, L, Q, LE, and GE) or a value of 1 (λ, γ, σ).

In comparison to random and lattice networks, there were significant differences across all metrics, C (*F* (2,339) = 1,284, *p* < 0.001), L (*F* (2, 339) = 170, *p* < 0.001), Q (*F* (2, 339) = 663, p < 0.001), LE (*F* (2, 339) = 708, *p* < 0.001), and GE (*F* (2,339) = 54.68, *p* < 0.001), with all *post hoc* analyses finding all between network differences significant at *p* < 0.001. Additional, maintenance of small-world topology was observed with σ (*t* (113) = 39.89, *p* < 0.001) significantly above 1.0. Finally, network measures did not differ between infants with high or low amounts of motion censoring (*p* > 0.140 following correction, Supplementary Table S2) (see Figure 1).

### 3.2. Economy of preterm networks

**Figure 2:**
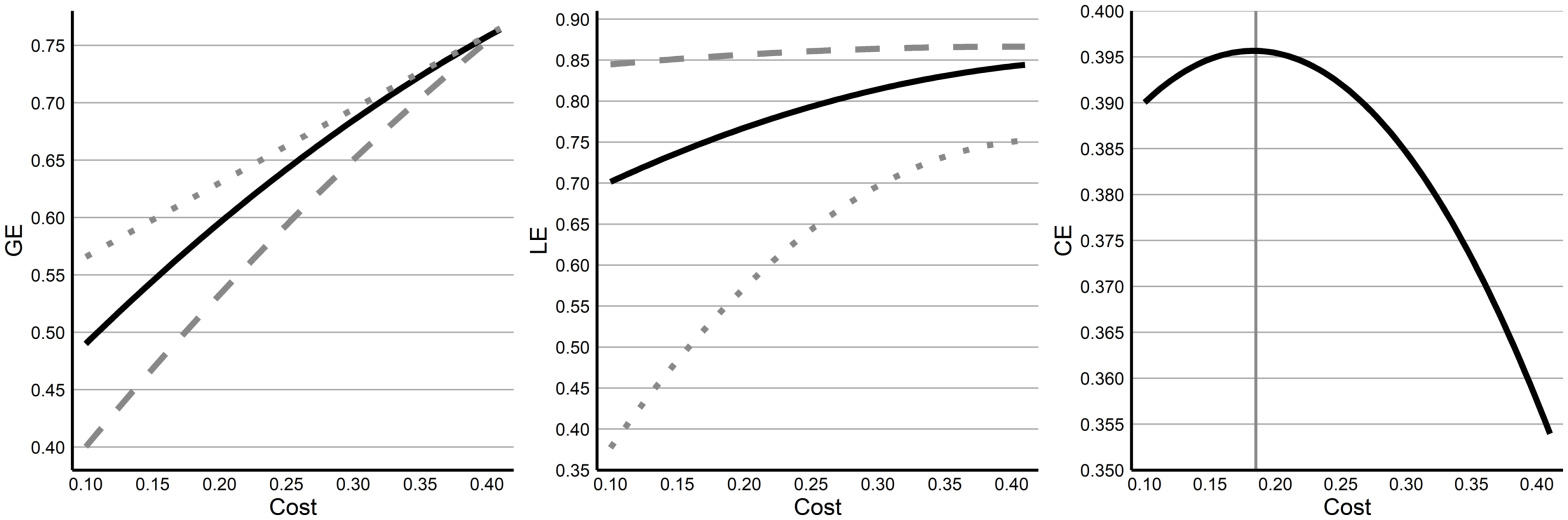
Economy and efficiency across costs. Across cost thresholds, GE and LE are shown compared to their random (dotted) and lattice (dashed) network values showing a decreasing effect with increasing cost (higher thresholding). Both GE and LE sit directly between values for their random and lattice network values. CE is then displayed across costs with a clear inversion point at a cost of 0.19.

Next, efficiency and cost for preterm networks were evaluated. Across costs, and consistent with the findings from prior comparisons, GE and LE both exhibit clear separation from their random and lattice network values, although near the top costs of 0.40, differences between GE networks decrease to negligible differences (Figure 2). Finally, while examining CE across all costs, no clear inflex point was observable across the costs examined, however, within the assessed values the highest cost efficiency was at 0.19, where CE was 0.3966.

### 3.3. Network changes across postmenstrual ages

**Figure 3:**
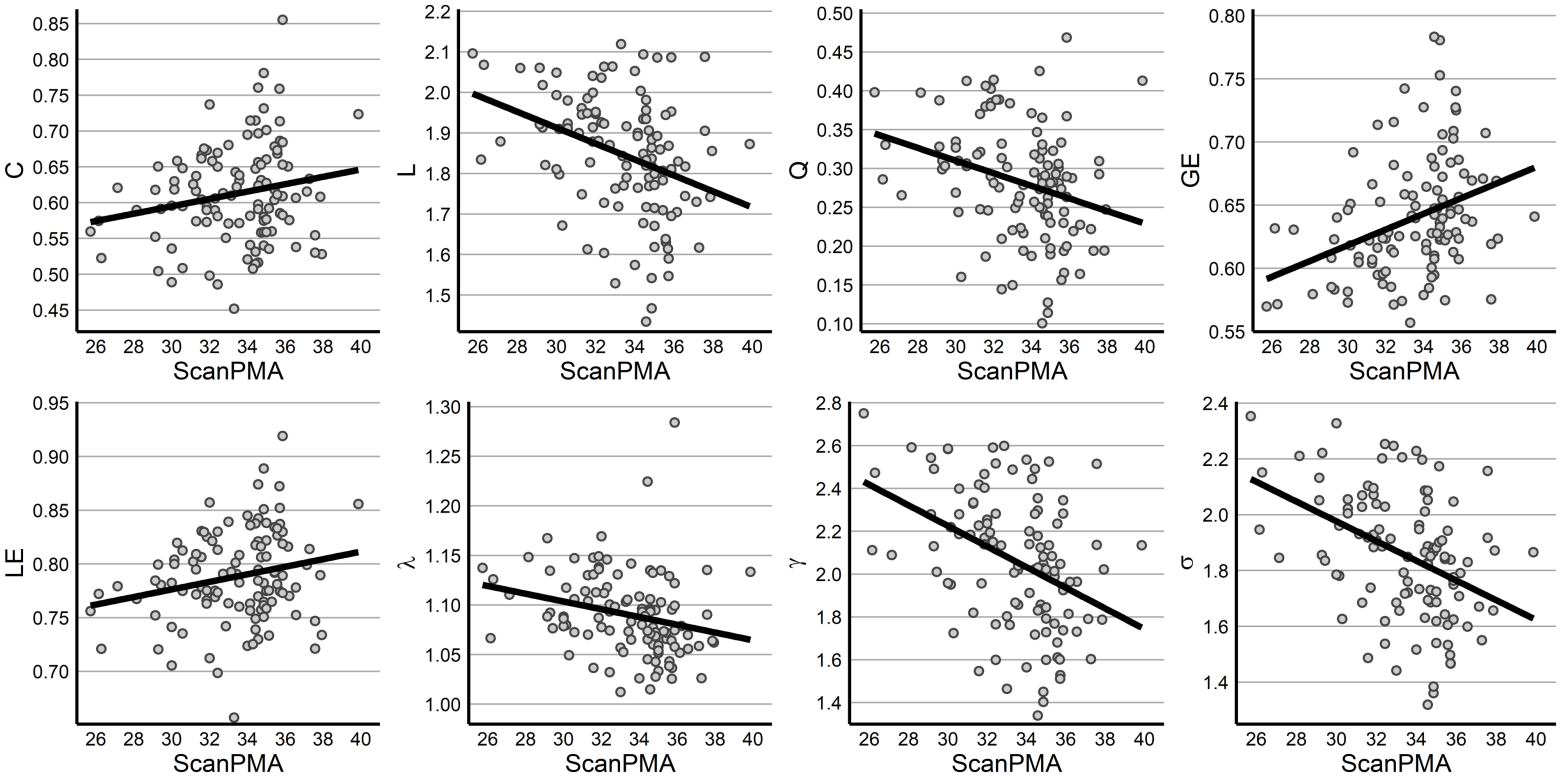
Graph properties across postmenstrual age. All graph metrics are displayed across PMA, with all significant relationships following FDR correction.

Finally, the impact of PMA, number of *ex-utero* weeks, and sex were evaluated across metrics using a linear mixed effects model to control for repeated measures from some infants. Following FDR correction, increasing PMA was associated with increased GE (*p* = 0.001) and decreasing L (*p* = 0.001), Q (*p* = 0.005), γ (*p* < 0.001), and σ (*p* < 0.001). Both C and LE exhibited increasing values while λ decreased with PMA but were only trending at *p* < 0.10 following correction (Table 2; Figure 3). Across all measures, however, sex was not significantly associated with network metrics.

**Table 2:** Changes in network metrics across preterm development.

	r^2^_adj_	PMA at scan			Sex (Male)		
		β	se	*p*	β	se	*p*
C	0.039	0.0052	0.0025	0.0453^b^	0.0038	0.0131	0.7740
L	0.116	−0.0195	0.0051	0.0007^a^	−0.0040	0.0280	0.8846
Q	0.082	−0.0080	0.0026	0.0047^a^	0.0077	0.0140	0.5816
GE	0.122	0.0062	0.0016	0.0006^a^	0.0021	0.0086	0.8057
LE	0.048	0.0035	0.0015	0.0280^b^	0.0026	0.0081	0.7461
λ	0.047	−0.0034	0.0014	0.0204^b^	−0.0011	0.0085	0.8941
γ	0.163	−0.0412	0.0104	0.0001^a^	0.0117	0.0554	0.8339
σ	0.167	−0.0353	0.0075	0.0001^a^	0.0128	0.0401	0.7503

a Significant at *p* < 0.05 following FDR correction.

b Significant at *p* < 0.10 following FDR correction.

## 4. Discussion

Our findings extend upon knowledge of healthy fetal and term-born neonatal brain development by characterizing differences in how the brain functionally organizes in *ex-utero* preterm infants. First, we observed a small-world topology across infants and PMA with both locally and globally efficient networks in the cohort, consistent with *in-utero* development during the second and third trimesters. Departing from prior findings in fetal development, however, we observed that infants born V/EPT exhibit significant increases in GE, and decreases in L, Q, λ, γ, and σ with increasing PMA, as well as trending decreases in C and LE. These results suggest that, while efficient small world topologies are preserved in *ex-utero* preterm infants relative to *in-utero* fetuses, there are notable changes in the developing functional network structure, characterized by differences in network segregation and integration resulting from the stressors of earlier-than-typical extrauterine exposures.

Consistent with prior work in fetuses and young children, we observe a preserved small-world topology across all individuals (Huang et al., 2015; Bouyssi-Kobar et al., 2019; De Asis-Cruz et al., 2021). We also observe cost effective network organizations across postmenstrual age, with clear evidence of locally and globally efficient networks. These results suggest that a fundamental network topology of a series of tightly connected and well-integrated regions (Humphries and Gurney, 2008) is preserved for *ex-utero* preterm infants relative to *in-utero* fetuses, and that preterm birth does not significantly alter functional network structure in a manner that removes small world characteristics which are also observed in *in-utero* fetuses and term born neonates.

Across postmenstrual age, we observe a clear increase in network integration with increasing age – exhibited as increased GE and decreased L. Conversely, network segregation is less consistent across metrics. Decreasing Q with PMA is indicative of decreasing segregation, and although C and LE are only marginally significant, their trending increases suggest increasing segregation. Moreover, we also observe a general decrease in small world properties (decreasing σ) with increasing age. The observed trajectories of increasing network segregation metrics of C and LE, while Q decreases with PMA differ from prior findings with similarly aged *in-utero* fetuses – but are more consistent with early postnatal development in term-born infants. Within the context of postnatal development, these results are consistent with prior literature on network development in the early-life period of term born infants. Namely, from the neonatal through preadolescent period, there are significant increases in integration and decreases in segregation, with consistent maintenance of small-world topology (Huang et al., 2015). During the neonatal and early childhood periods, the brain is undergoing structural and functional development including refining white matter pathways though both myelination and pruning of structural connections (Innocenti and Price, 2005) as well as the formation of more mature functional networks (Gilmore et al., 2018). These findings in infancy following term-age birth suggest that V/EPT infants during the preterm period are experiencing an accelerated trajectory of functional maturation typically observed following full-term birth.

While the developmental trajectories of V/EPT infants are directionally consistent with postnatal development of infants born at term, the trajectories noticeably deviate from *in-utero* development during this period. Namely, *in-utero* fetuses experience decreasing segregation across gestational age leading up to birth, marked by decreases in Q, LE, with a trending decrease in C, and no significant changes in integration (De Asis-Cruz et al., 2021). Although similar effects were observed within decreasing small-worldness. The differences are further compounded by work suggesting measures of segregation, C, Q, and LE are lower in V/EPT compared to term-born infants at TEA (Bouyssi-Kobar et al., 2019). Taken together, these data suggest that the typical trajectory in subsequently term-born infants follows a general decrease in segregation up to the time of birth, followed by increases in segregation and integration throughout early infancy and early childhood. Compared to developmental changes in the fetal and early postnatal period of term-born infants, our results suggest that the development of functional network architecture of V/EPT infants more closely mirrors the postnatal period rather than the fetal period, despite the greater disparity in postmenstrual age between V/EPT and term-born neonates. Therefore, the effects we are observing may suggest that extrauterine experience and exogenous influences may be preempting age-typical neurodevelopment leading to the previously reported differences between term and V/EPT infants and may be the target for neuroprotective strategies.

Despite our robust findings, there are some limitations that should be noted. First and foremost, while a smaller percentage of our sample has repeated scans, the majority only have a single viable scan. As a result, our findings are largely cross-sectional. Future work is required to examine longitudinal effects for individual infants across the period from birth to term equivalent age to fully account for individual differences across development. Second, when interpreting these results within the literature on typical development should be done cautiously. While fetuses are more closely aligned in maturation, acquisition and processing of fetal data often is quite different from ex utero scanning as there are additional artifacts and concerns with signal changes due to capturing images *in utero*. Conversely, ex utero healthy neonatal data can be collected and processed similarly, but these infants are significant older by definition. Therefore direct comparisons between both groups in ex utero preterm infants should be performed judiciously. Moreover, as a clinical population, a more systematic investigation is needed to disentangle the manner of extrauterine exposures that play a role in the observed differences as well the long-term significance is of the observed alterations in functional brain architecture – especially for those who go on to experience difficulties during infancy and toddlerhood compared to those to exhibit a more typical developmental trajectory. Third, the high rates of perinatal brain injury may further complicate this concern, however, in our sample we did not observe any differences between those with no injury and those with mild injury (Supplementary Table S1), although the results are likely to be different in infants with more significant injury Follow-up investigations should more finely examine the impact of injury types on network topography. Fourth, it should be noted that we utilized an anatomical parcellation of the brain to calculate our network metrics. Prior work in adults has suggested that there is intersubject variability in brain structure (Gordon et al., 2017). Future work should examine the impact of functional versus structural parcellations methods in neonates. Additionally, due to the particular vulnerability of the younger infants in the study, we elected to utilize a 1.5 T scanner out of an abundance of caution for their clinical state. As a result, there is a greater amount of noise possible in our data, and subsequent studies should examine the possible impact of field strength on our findings.

This study represents the first investigation of graph network architecture during the extra-uterine life of V/EPT infants prior to reaching term-equivalent age. Our findings suggest that *ex-utero* development of functional brain networks exhibit age dependent changes in functional brain organization. The observed changes are consistent with changes in *ex-utero* network development observed in term-born infants but differ from what has been observed in fetuses of similar ages while *in-utero*. These results offer the first quantification of how preterm developmental trajectories may be altered even prior to term age leading to future neurodevelopmental deficits and therefore provides a target for early novel neuroprotective strategies to improve outcomes.

## Data availability statement

The raw data supporting the conclusions of this article will be made available by the authors, without undue reservation.

## Ethics statement

The studies involving humans were approved by Children’s National Hospital Institutional Review Board. The studies were conducted in accordance with the local legislation and institutional requirements. Written informed consent for participation in this study was provided by the participants’ legal guardians/next of kin.

## Author contributions

KC, JD, and CL made substantial contributions to the planning and conceptualization of the project. ES and JM contributed to the collection and quality checking of data. KC and JD conducted the statistical analyses for the manuscript. KC, JD, SB, NA, JM, ES, AP, and CL all offered significant input and contributed to the preparation, editing, and reviewing of the final manuscript. All authors contributed to the article and approved the submitted version.

## Funding

This work was supported by the NIH Eunice Kennedy Shriver National Institute of Child Health and Human Development at the National Institutes of Health (T32-HD098066 to KC, U54-HD090257 & R01-HD099393 to CL).

## Conflict of interest

The authors declare that the research was conducted in the absence of any commercial or financial relationships that could be construed as a potential conflict of interest.

## Publisher’s note

All claims expressed in this article are solely those of the authors and do not necessarily represent those of their affiliated organizations, or those of the publisher, the editors and the reviewers. Any product that may be evaluated in this article, or claim that may be made by its manufacturer, is not guaranteed or endorsed by the publisher.
