# Supplementary material for: Ex-utero third trimester developmental changes in functional brain network organization in infants born very and extremely preterm
Source: Front Neurosci. 2023 Sep 1;17:1214080. doi: 10.3389/fnins.2023.1214080 (PMC10502339; doi:10.3389/fnins.2023.1214080)
Supplement: Supplementary file 1 [file Data_Sheet_1.docx]

**Supplemental Information**

**Ages at birth and scan for included participants**


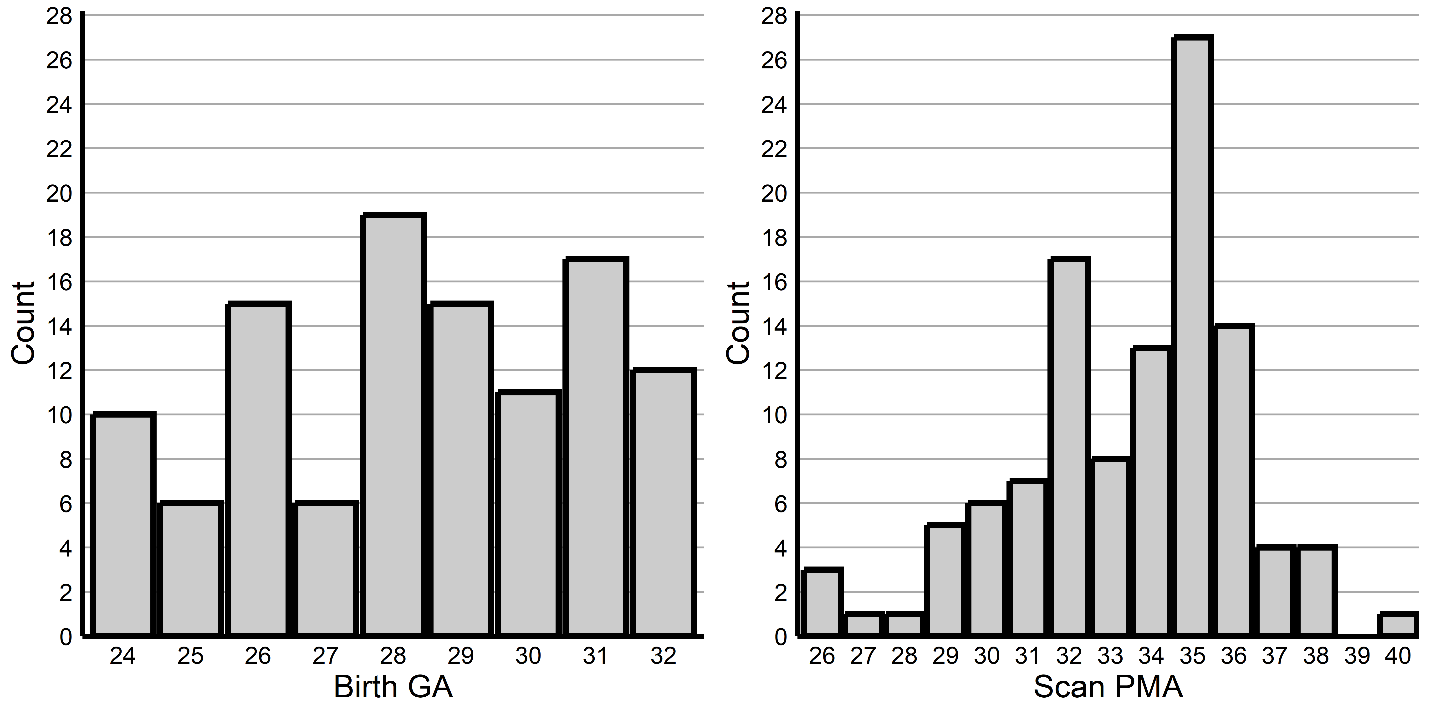


*Supplemental Information Figure 1*

*Distribution of included participants by gestational age (GA) at birth and postmenstrual age (PMA) at time of scan.*

**Impact of injury**

Control analyses were conducted to compare the impact of injury on network metrics. First, groups were divided into a no injury (n=67) and mild injury (n=44) group based on their kidokoro scores. There was no differences for either GA at birth *t*(109)=0.9727, p=.333 or PMA at scan *t*(109)=0.389, p=.698 between groups (SI Figure 2)

To assess the impact of injury on network metrics, linear mixed effect models were performed independently for each metric. Given the observed impact of PMA at scan on network metrics, PMA was included in the model and repeated measures were controlled for. Following multiple comparisons correction, no differences were observed between the no and mild injury groups on any of the metrics (p_corr_ < .140, SI Table 1)


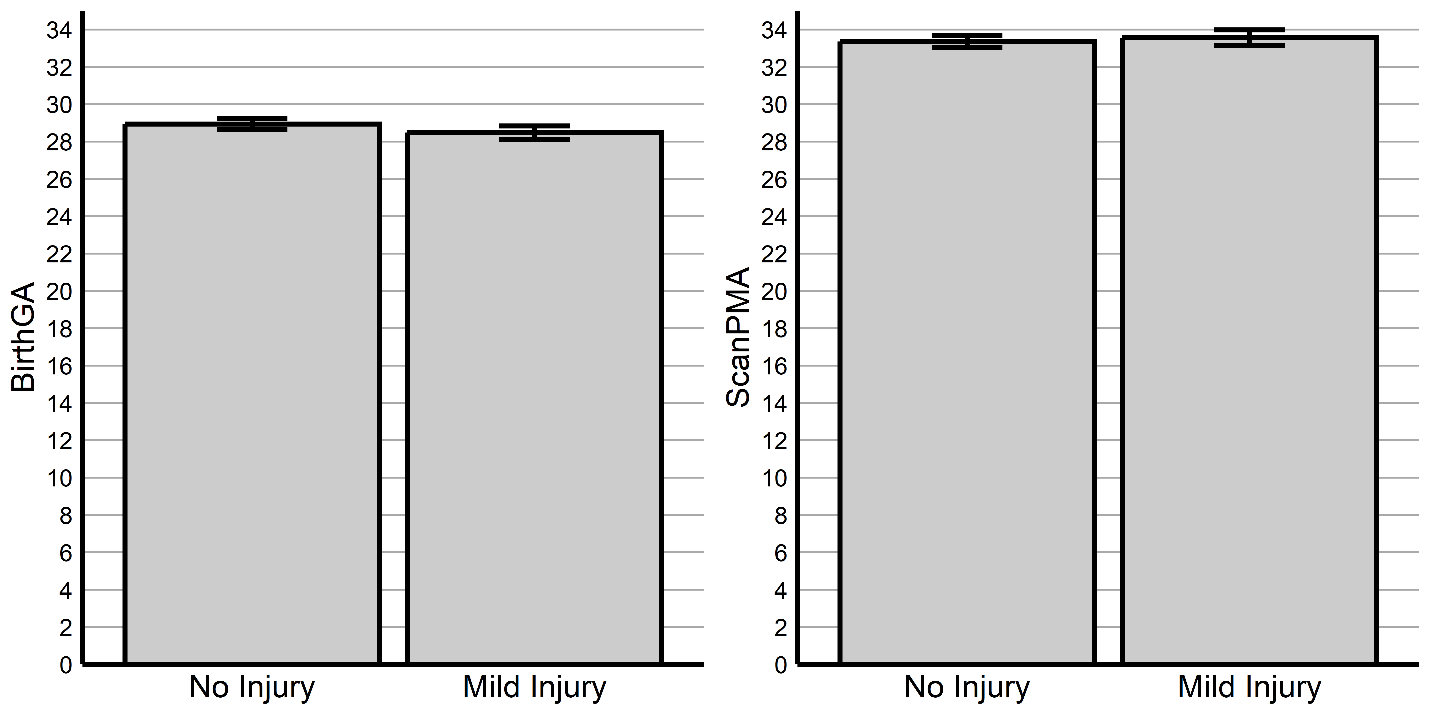


*Supplemental Information Figure 2*

*No significant differences were observed in gestational age (GA) at birth and postmenstrual age (PMA) at time of scan between infants with no injury and those with mild injury.*

| SI Table 1 Linear mixed model effect of injury | | | | |
| --- | --- | --- | --- | --- |
|  | Injury (None) | | | |
|  | β | se | p | p_corr_ |
| C | 0.0008 | 0.0132 | .5308 | .5308 |
| L | -0.0592 | 0.0276 | .0351 | .1404 |
| Q | -0.0132 | 0.0141 | .3538 | .4043 |
| GE | 0.0160 | 0.0085 | .0641 | .1709 |
| LE | 0.0079 | 0.0082 | .3367 | .4043 |
| λ | -0.0912 | 0.0556 | .1024 | .2048 |
| γ | -0.0482 | 0.0405 | .2371 | .3793 |
| σ | -0.0195 | 0.0083 | .0213 | .1404 |
| * Significant at <.05 following FDR Correction  ^†^ Significant at <.10 following FDR Correction | | | | |

**Impact of motion censoring**

Control analyses were conducted to compare the impact of motion censoring on network metrics. All scans were divided into a low censor and a high censor group based on the number of available volumes following scrubbing. Groups were determined based on a median split of the total number of available volumes (median = 193 volumes). Two infants with the median value were excluded, yielding a low censor group (n=55) with an average of 245.2 (33.4) volumes and a high censor group (n=54) with an average of 154.9 (22.0) volumes. Consistent with prior literature on motion in younger born infants, we observed that the high censor group was significantly younger at birth *t*(107)=3.764, p<.001 as well as at scan *t*(107)=2.475, p=.0149, compared to the low censor (SI Figure 3).

To assess the impact of censoring on network metrics, linear mixed effect models were performed independently for each metric. Given the observed impact of PMA at scan on network metrics, PMA was included in the model and repeated measures were controlled for. Following multiple comparisons correction, no differences were observed between the low and high censor groups on any of the metrics (p_corr_ < .1536, SI Table 2)


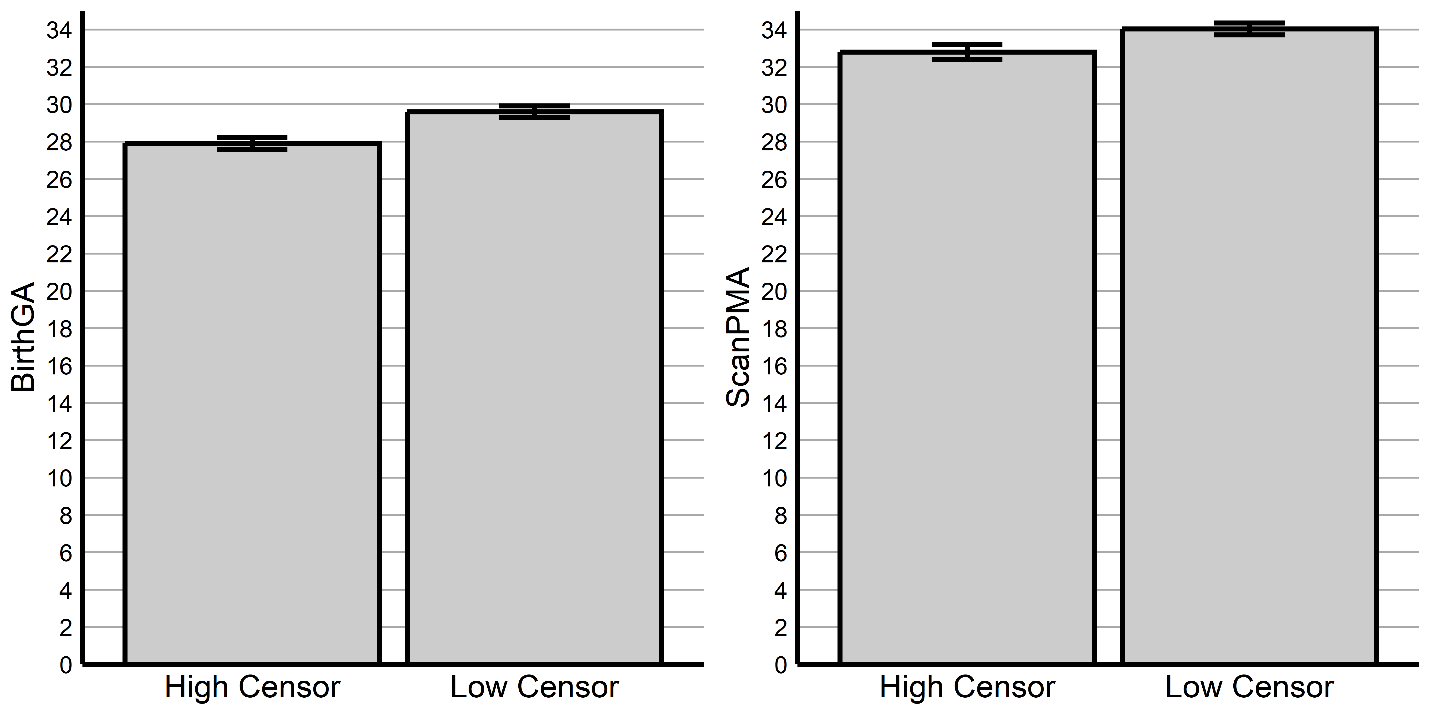


*Supplemental Information Figure 3*

*Significant differences were observed in gestational age (GA) at birth and postmenstrual age (PMA) at time of scan between infants, with the group having a high number of volume censored being significantly younger at birth and scan compared to infants with less censoring.*

| SI Table 2 Linear mixed model effect of motion censoring | | | | |
| --- | --- | --- | --- | --- |
|  | Motion Censor (Low) | | | |
|  | β | se | p | p_corr_ |
| C | 0.0189 | 0.0143 | .1720 | .1841 |
| L | -0.0575 | 0.0276 | .0484 | .1536 |
| Q | -0.0193 | 0.0140 | .1841 | .1841 |
| GE | 0.0186 | 0.0086 | .0413 | .1536 |
| LE | 0.0122 | 0.0083 | .1526 | .1841 |
| λ | -0.1060 | 0.0559 | .0703 | .1536 |
| γ | -0.0750 | 0.0405 | .0768 | .1536 |
| σ | -0.0117 | 0.0079 | .1530 | .1841 |
| * Significant at <.05 following FDR Correction  ^†^ Significant at <.10 following FDR Correction | | | | |
